# *NUCOME*: A comprehensive database of nucleosome organization referenced landscapes in mammalian genomes

**DOI:** 10.1186/s12859-021-04239-9

**Published:** 2021-06-13

**Authors:** Xiaolan Chen, Hui Yang, Guifen Liu, Yong Zhang

**Affiliations:** Institute for Regenerative Medicine, Shanghai East Hospital, Shanghai Key Laboratory of Signaling and Disease Research, Frontier Science Center for Stem Cell Research, School of Life Science and Technology, Tongji University, 1239 Siping Road, Shanghai, 200092 China

**Keywords:** Nucleosome, Database, Transcriptional regulation, MNase

## Abstract

**Background:**

Nucleosome organization is involved in many regulatory activities in various organisms. However, studies integrating nucleosome organization in mammalian genomes are very limited mainly due to the lack of comprehensive data quality control (QC) assessment and uneven data quality of public data sets.

**Results:**

The *NUCOME* is a database focused on filtering qualified nucleosome organization referenced landscapes covering various cell types in human and mouse based on QC metrics. The filtering strategy guarantees the quality of nucleosome organization referenced landscapes and exempts users from redundant data set selection and processing. The *NUCOME* database provides standardized, qualified data source and informative nucleosome organization features at a whole-genome scale and on the level of individual loci.

**Conclusions:**

The *NUCOME* provides valuable data resources for integrative analyses focus on nucleosome organization. The *NUCOME* is freely available at http://compbio-zhanglab.org/NUCOME.

**Supplementary Information:**

The online version contains supplementary material available at 10.1186/s12859-021-04239-9.

## Background

The nucleosome is the fundamental unit of eukaryotic chromatin and is involved in regulatory activities through interactions with DNA binding proteins, including regulatory factors, chromatin remodelers, histone chaperones and polymerases [1]. Genome-wide nucleosome organization maps have been established in multiple species, and several consistent nucleosome positioning patterns of specific regulatory elements have been reported. For example, nucleosomes act as barriers to transcription factors (TFs) interacting with *cis*-regulatory elements. Nucleosome-free regions (NFR) and regularly spaced nucleosome arrays are strongly associated with transcription initiation [1–4]. Nucleosome remodelers are essential for nucleosome dynamics that remove, slide and reposition nucleosomes to overcome barriers and facilitate transcription initiation and elongation [3, 5–7]. The conserved nucleosome positioning patterns on transcription start sites (TSSs) and other regulatory elements highlight the importance of the nucleosome organization in regulatory activities.

Furthermore, previous studies have shown that tissue- and disease-specific nucleosome organization widely exists in the mammalian genomes and is involved in cell differentiation [8, 9], reprogramming [9, 10], tissue impairment [11–13] and diseases [14–16]. Most studies focus on identifying specific nucleosome organization features and associating these features with transcription activities or chromatin modifications. Cell type-specific nucleosome organization typically indicates the chromatin environment that involves distinct regulatory factors and cellular processes [17]. Therefore, nucleosome organization maps are critical for deriving a panoramic view regarding the chromatin structure, modification and their relationships in regulatory functions. However, compared to other types of regulatory landscapes, such as histone modification, DNA methylation and transcription factors, nucleosome organization has not been sufficiently explored.

There are two major difficulties that impede the exploration of the nucleosome organization and the regulatory function. First, nucleosomes occupy a large percentage of the genome, which requires fairly high sequencing depth for nucleosome organization maps in human and mouse. The sequencing coverage has been proven to influence data quality in our previous research [18]. The impact of sequencing coverage makes public nucleosome organization maps vary widely in terms of data quality. The second difficulty lies in the analysis method, which is also limited by the data quality. Currently, researchers usually illustrate nucleosome organization features by aggregating nucleosome organization profiles on a large number of genes or genomic regions, which ignores the difference between individual gene or genomic region. However, some studies have revealed that the heterogeneity of the nucleosome organization pattern universally exists in TSSs, transcription factor binding sites and histone modification regions [19, 20].

To systematically explore the regulatory function of the nucleosome, a comprehensive and dedicated database of nucleosome organization landscapes is urgently needed to manage, explore and analyze these data resources. MNase-seq is the most widely used technology for generating nucleosome organization maps [21, 22]. The large size of mammalian genomes is a major challenge in experiments that require a very high sequencing depth; thus, MNase-seq data analyses are complicated and time consuming. We established a comprehensive database named *NUCOME* (**Nuc**leosome **O**rganization Referenced Landscapes in **M**ammalian G**e**nomes) that organizes MNase-seq data sets and characterizes nucleosome organization. The *NUCOME* database solves the difficulties mentioned above using an integration strategy combining quality control (QC) assessment and pooling of samples. The followings are the three major features of the *NUCOME* database: (1) *NUCOME* manages and filters extensive MNase-seq data in human and mouse via a standardized analysis pipeline and QC metrics. (2) *NUCOME* provides high-quality nucleosome organization referenced landscapes for various human and mouse cell and tissue types, providing more reliable nucleosome organization information. (3) *NUCOME* provides a web interface containing multiple modules, including processed data download, an analysis module that quantifies informative nucleosome organization features in genomic regions, and search modules for displaying multiple nucleosome organization features among different cell or tissue types in an individual gene or loci manner. Currently, there are a few databases focusing on curating nucleosome organization maps. For example, the NucMap database [23] collects public data sets in various species without filtering data based on quality, while another study summarise nucleosome organization data resources [24]. Compared with the available database of nucleosome organization maps, *NUCOME* has advantage on data filtration based on QC, which not only exempts the users from redundant data selection but also verifies the reliability and accuracy of quantified nucleosome organization information.

## Construction and content

### Nucleosome organization referenced landscapes filtration based on QC assessments and pooling of samples

**Fig. 1:**
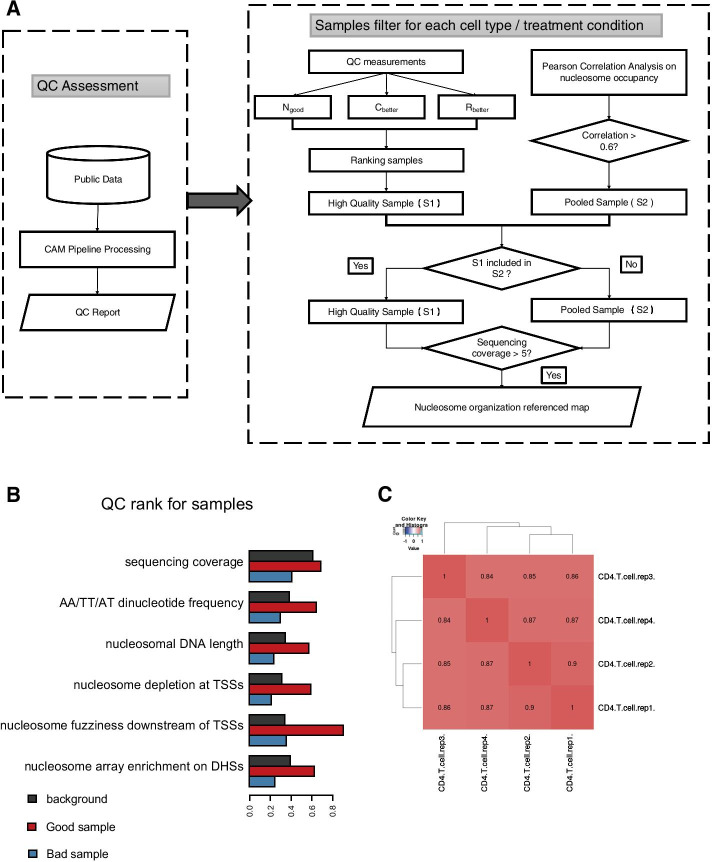
The filtration and pooling strategy of MNase-seq data*.* **a** The workflow of landscape selection that combines data filtration based on QC measurements with the pooling of samples. **b** An example of rank samples by QC measurements among samples in CD4+ T cells. The gray boxes represent the average QC measurement rank quantiles for all CD4+ T cells, the red boxes represent a sample with higher QC rank quantiles versus the average level, and the blue boxes represent a sample with lower QC rank quantiles versus the average level. **c**. An example of correlation analysis among samples in CD4+ T cells, the Pearson correlation coefficient was labeled in boxes

*NUCOME* focuses on filtering qualified human and mouse MNase-seq data. We collected 509 available data sets from the Gene Expression Omnibus (GEO; https://www.ncbi.nlm.nih.gov/geo/) and downloaded raw data sets from the Sequence Read Archive (SRA; http://www.ncbi.nlm.nih.gov/sra). The data source comprises 69 kinds of mouse cell or tissue types and 33 kinds of human cell or tissue types (Additional file 1: Table S1, S2). All data was processed with a standardized analysis pipeline named CAM, which we developed previously [18], accompanying with QCs measurements specific for MNase-seq data. The following six QC measurements were introduced: sequencing coverage, AA/TT/AT dinucleotide frequency, nucleosomal DNA length, nucleosome depletion at TSSs, nucleosome fuzziness downstream of TSSs, and enrichment of well-positioned nucleosome arrays at DNase hypersensitive sites (DHSs). The QC measurements show that the public MNase-seq data quality vary greatly among samples (Additional file 1: Figure S1). To exempt users from confusion in selecting high-quality data among samples, we determined nucleosome organization referenced landscapes based on a strategy that combined quality ranking with pooling of samples (Fig. 1a). We ranked the samples from the same cell or tissue type based on three summarized QC indicators (N_good,_ C_better_ and R_better_), which are calculated from six QC measurements (Fig. 1b) as follows. N_good_ is the first rank indicator, which represents the number of QC measurements that evaluated as ‘Pass’ for each sample. C_better_ is the second rank indicator, which represents the number of QC measurements that have higher prioritization rank than average quality of samples from the same cell type. R_better_ is the third rank indicator, which represents the sum of rank quantiles of the six QC measurements for each sample. The final ranking orders for the samples were dependent on N_good_, C_better_ and R_better_ successively, and the top sample of each cell or tissue type was selected as the ‘High Quality Sample’ (Fig. 1a). We also pooled samples with a high correlation to generate ‘Pooled Sample’ (Fig. 1c). ‘High Quality Sample’ was defined as the referenced nucleosome organization landscape for each cell type and treatment condition, unless the ‘High Quality Sample’ was included in the samples which were used to build the ‘Pooled Sample’ (see Methods for details).

### Nucleosome organization features on TSSs and DHSs

We designed multiple informative nucleosome organization features on TSSs and DHSs in an individual gene or genomic region manner so that the heterogeneity among different genes or genomic regions can be displayed. The high-quality nucleosome organization referenced landscapes have two major benefits. First, the QC assessment confirms the reliability of these nucleosome organization features on individual gene or DHS. Then, the filtration and pooling strategy made the referenced landscapes more comparable due to the high-quality and high-coverage characteristics.

## Utility and discussion

We integrated the nucleosome organization referenced landscapes and nucleosome organization features on TSSs and DHSs in an available database named *NUCOME* (http://compbio-zhanglab.org/NUCOME). The nucleosome organization referenced landscapes allow users to conduct their own analyses without difficulties in data preprocessing and QC. Nucleosome organization features on TSSs and DHSs are displayed to explore the difference between cell or tissue types.

**Fig. 2:**
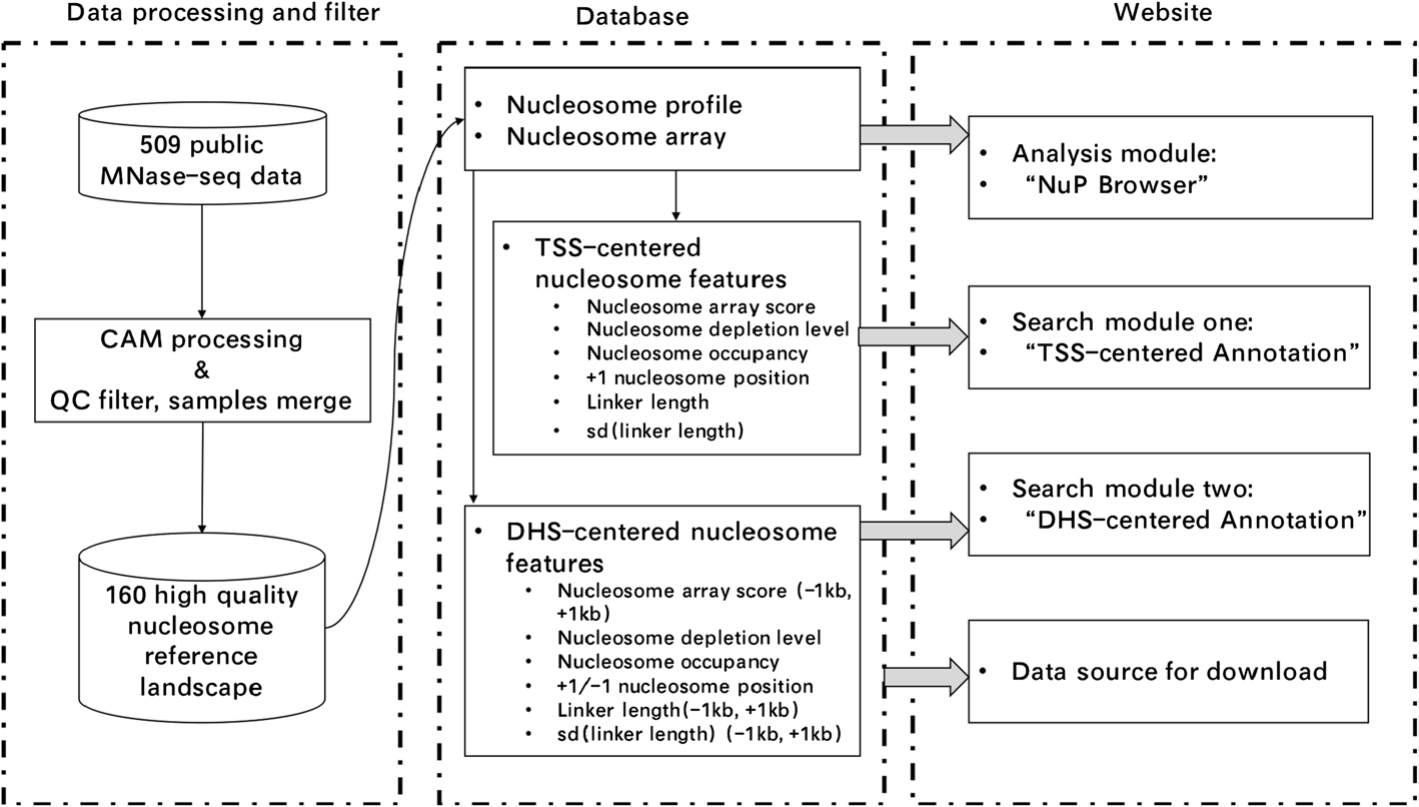
The content and structure of the *NUCOME* database

*NUCOME* includes three analysis or search modules (Fig. 2), i.e., (a) ‘NuP Browser’ (nucleosome positioning browser), (b) ‘TSS-centered Annotation’ and (c) ‘DHS-centered Annotation’. In addition, *NUCOME* also includes a ‘Download’ module for providing access to download standardized processed files or text files that store nucleosome organization features on TSSs and DHSs.

The interpretations of modules are briefly explained below. For the analysis and search modules, we provide examples for their usage and application. The first example explores the expanded usage of MNase-seq data for predicting TF binding events to illustrate interaction mechanism of nucleosome organization with other regulatory factors. The second example illustrates the correlation between nucleosome organization and transcription activity among samples for individual gene. The third example shows nucleosome organization features on individual DHS among several cell types indicating the difference in DNA accessibility.

### *NUCOME* provides qualified nucleosome organization referenced landscapes

**Fig. 3:**
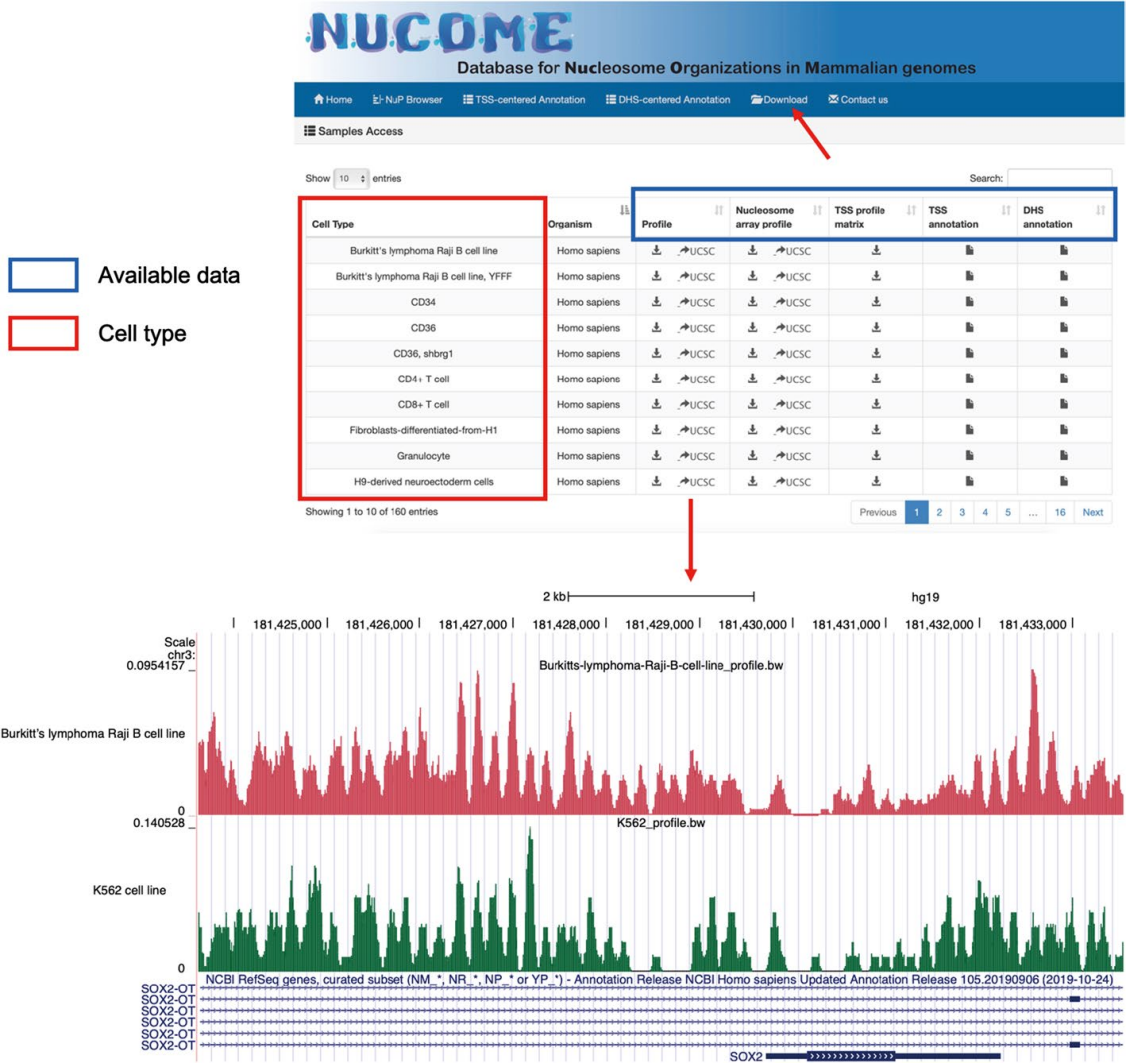
The available data source of the *NUCOME* database

*NUCOME* offers a convenient approach for users to download files with standardized processing in the ‘Download’ module (Fig. 3). The bigWig files for nucleosome occupancy and nucleosome array allow users to conduct their analyses on any genomic region. Other files that store nucleosome organization features in advance are valuable for users to explore the regulatory function of nucleosome organization in individual gene or DHS. In addition, access to visualization of nucleosome organization profiles in the UCSC browser is also provided in this module.

### Nucleosome organization improves transcription factor binding site prediction

**Fig. 4:**
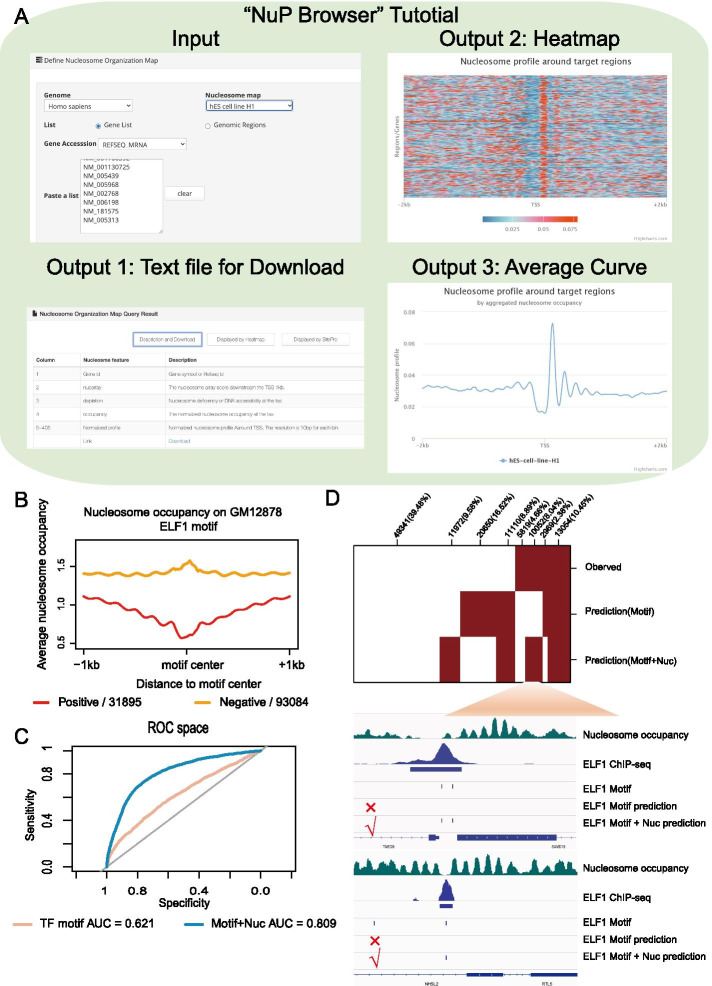
The interpretation and application example of the ‘NuP Browser’ module. **a** The operation and results exhibition of the ‘NuP Browser’ module. **b** The average nucleosome profile of positive samples or negative samples in ELF1 binding prediction model in the GM12878 cell line. The red line represents the ELF1 motif hits overlapping with ChIP-seq peaks. The orange line represents the ELF1 motif hits that not overlap with ChIP-seq peaks. **c** The ROC curve and AUC score for the ELF1 prediction model. The orange line represents the prediction performance of the model with only motif scores. The blue line represents the prediction performance of the model that includes nucleosome organization features. **d** The practical TF binding status and prediction results of ELF1 in the GM12878 cell line. Two examples failed to be recognized by the prediction model with only motif scores but were recognized after nucleosome organization features introduction

The ‘NuP Browser’ module provides users with a friendly and flexible platform to query nucleosome organization information in any gene or genomic region. The ‘NuP Browser’ module quantifies multiple nucleosome organization features describing the local chromatin structure, including nucleosome occupancy, nucleosome array score, nucleosome depletion level and nucleosome profile. The text format output allows users to perform nucleosome positioning analyses without encountering difficulties in data processing and filtering. The ‘NuP Browser’ also displays the analysis results by drawing the average curve and heatmap of the nucleosome profile on the target regions (Fig. 4a).

Previous studies have confirmed that nucleosome positioning patterns around TFs may indicate the regulatory function of TFs and play a predictive role in gene expression [19]. Here, we explored the ability to predict TF binding in vivo by introducing informative nucleosome organization. We used logistic linear regression to match the binding status of TFs in 36 ChIP-seq data sets of TFs covering 6 different cell types in human (Additional file 1: Table S3) by scoring the motif score of DNA sequence and nucleosome organization features for those sites. Then, we performed a receiver operating characteristic (ROC) curve analysis to evaluate the performance of the prediction based on AUC scores (see Methods for details). Take for example, the average curves show that ELF1 prefers to bind to locations with lower nucleosome occupancy and higher nucleosome depletion levels in GM12878 cell line (Fig. 4b). Nucleosome organization features significantly improved the AUC score (Fig. 4c). Nucleosome organization features can distinguish positive and negative motif hits better than motif scores (Fig. 4d). To summarize the overall performance of nucleosome organization in TF binding site prediction, we trained two prediction models for each TF in the specific cell type respectively, and compared the prediction power when nucleosome organization features were included. The results demonstrate that the AUC scores improve universally after including the nucleosome organization features in the prediction model (Additional file 1: Figure S2A). The improvements may result from the effect of nucleosome organization on chromatin accessibility for TF binding. The improvements are not equal in all TFs, and the TFs can be classified into the Improve_G and Improve_S groups, with great or slight improvement of prediction power by adding nucleosome organization features (Additional file 1: Figure S2B). We found that the improvements in TF binding prediction were significantly negatively correlated with the intrinsic DNA motif prediction contribution (Additional file 1: Figure S2C). Besides, the introduction of nucleosome organization features in the TF binding site prediction model results in universal increases in the sensitive, precision, F1 score and AUC score of the model, and partial improvements in the specificity and FPR (Additional file 1: Figure S2D). Taken together, these results demonstrate that nucleosome organization information derived from the ‘NuP Browser’ module can be widely used to elucidate the transcription regulatory program in mammals.

### Nucleosome organization regulates transcription activity

**Fig. 5:**
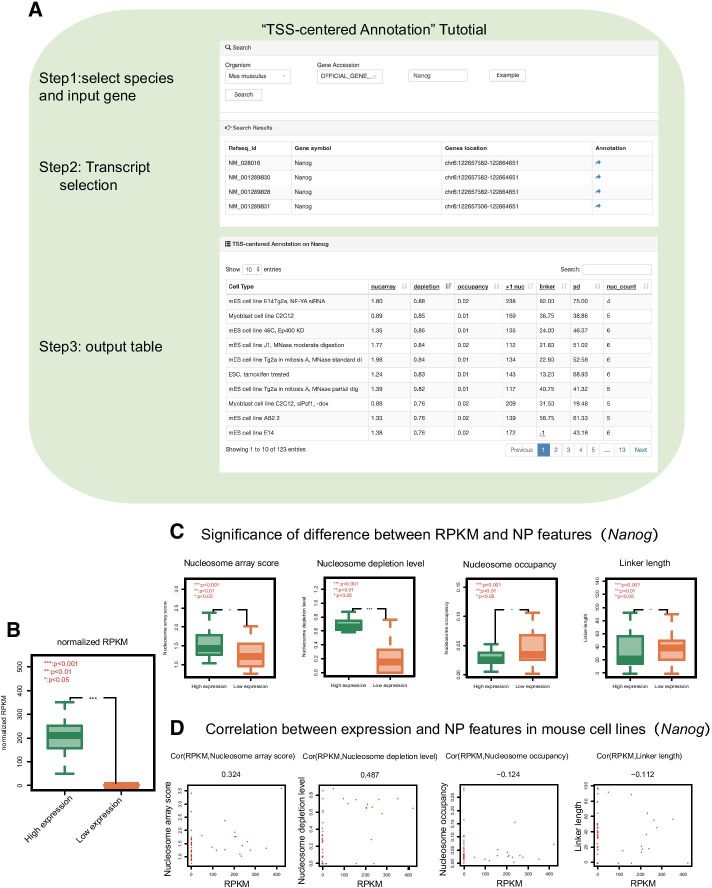
The interpretation and application example of the ‘TSS-centered Annotation’ module. **a** The operation and results exhibition of the ‘TSS-centered Annotation’ module. **b** Classification of the 47 samples into high expression level and low expression level by RPKM of the *Nanog* gene in mouse. **c** The differences in several nucleosome organization features between the two classes of samples. The p-value was calculated by the rank-sum test. **d** The correlation between gene expression level and nucleosome organization features. The Pearson correlation coefficient is marked above the scatter plots. The nucleosome organization features on **c** and **d** include nucleosome array scores downstream of the TSS 1 kb bin, nucleosome depletion level on the TSS, nucleosome occupancy on the TSS and linker length downstream of the TSS 1 kb bin

The ‘TSS-centered Annotation’ module allows users to request multiple nucleosome organization features on their target gene. The search results are displayed in a table containing nucleosome depletion level, nucleosome array score, nucleosome occupancy, + 1 nucleosome position, linker length, standard deviation of linker length and the number of nucleosomes downstream 1 kb of the TSS among different cell or types (Fig. 5a, see Methods for details).

The relationship between nucleosome organization and transcription activity has been illustrated by many previous studies in the strategy of aggregate nucleosome organization profile on genes classified by expression level in a specific cell type. Previous studies showed that highly expressed genes tend to have apparent nucleosome free regions (NFRs) on TSSs, corresponding to lower nucleosome occupancy on TSSs. Regarding the canonical nucleosome positioning pattern, highly expressed genes showed more regularly spaced nucleosomes downstream of the TSSs [25]. Take the *Nanog* gene for example, the search results show that the nucleosome depletion level in pluripotent cells tended to be higher than that in other cell types (Fig. 5a), coinciding with the highly transcriptional activity of the *Nanog* gene in pluripotent cells. Whether the above correlations exist on individual gene among various cell types can be explained by the nucleosome organization features provided by the‘TSS-centered Annotation’ module. In mouse, we performed correlation analysis between the expression level of *Nanog* and nucleosome organization features on TSS among 47 kinds of cell types or treatment conditions (Fig. 5b–d). The nucleosome array score and nucleosome depletion level show significantly positive correlations with the expression level, which corresponds with the previous knowledge. However, the nucleosome occupancy and linker length show relatively lower negative correlations with the expression level. In this case, we conclude that the previous knowledge could not completely explain the relationship between nucleosome organization and transcription activity at the individual gene level. This search module allows users to deeply explore the features of nucleosome-mediated regulation among different cell types or treatment conditions in a manner of individual gene.

### Nucleosome organization influences genome accessibility and transcription factor binding

**Fig. 6:**
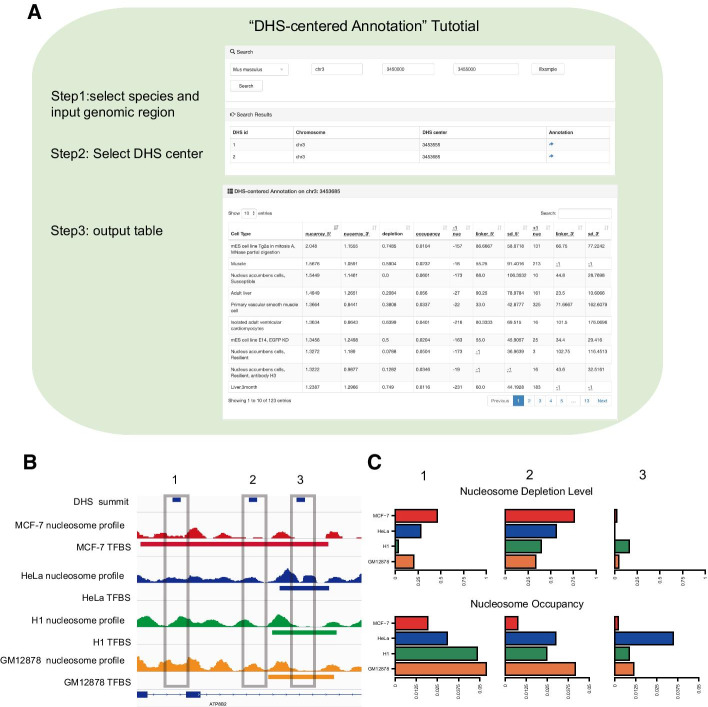
The interpretation and application example of the ‘DHS-centered Annotation’ module. **a** The operation and results exhibition of the ‘DHS-centered Annotation’ module. **b** The nucleosome occupancy profile and TF binding status for four cell lines displayed with an IGV browser. **c** The nucleosome depletion level and nucleosome occupancy on each DHS summit among four cell lines. The box colors correspond to the color in **b**, representing different kinds of cell lines

The ‘DHS-centered Annotation’ module is similar to the ‘TSS-centered Annotation’ in terms of nucleosome organization features, operation and result exhibition. Compared with the ‘TSS-centered Annotation’ module, ‘DHS-centered Annotation’ provides more features on individual DHSs among different cell types or treatment conditions (Fig. 6a, see Methods for details).

Here, we explored the relationship between nucleosome organization and DNA accessibility and transcription factor binding events by integrating nucleosome organization features provided by ‘DHS-centered Annotation’ and public ChIP-seq data for TFs. In the case of the first two DHSs, the MCF-7 specific TF binding events are accompanied by higher nucleosome depletion level and lower nucleosome occupancy than other cell lines. In another case, the DHS is occupied by TFs in all cell lines, the nucleosome occupancy signals support that the DNA is highly accessible in these cell lines. The nucleosome depletion levels show contrary results (Fig. 6b,c). which can be explained by the influence of neighboring nucleosomes and the width of the nucleosome free region. The users are able to explore the regulatory function of nucleosome organization in local chromatin structure and DNA–protein interactions through this module.

## Conclusion

*NUCOME* is a comprehensive database that organizes the qualified data sources of MNase-seq data and provides nucleosome organization referenced landscapes of various cell and tissue types in human and mouse. *NUCOME* provides modules in a web interface for three major function: (1) querying nucleosome organization information in any genomic region and providing text format outputs for users’ downstream analyses, (2) querying nucleosome organization features on individual gene or DHS among various samples, and (3) querying data sources for download. Given that nucleosome organization participates in various regulatory activities, and that *NUCOME* is a comprehensive database that organizes the nucleosome organization referenced landscapes supervised by QC measurements, it is a valuable resource that can be used to elucidate the panoramic view of transcription regulatory programs in human and mouse.

## Methods

### Nucleosome organization referenced landscapes filtration

*NUCOME* filters nucleosome organization samples based on six quality control measurements. The definitions of sequencing coverage, AA/TT/AT dinucleotide frequency, nucleosomal DNA length, nucleosome depletion at TSSs and enrichment of well-positioned nucleosome arrays at DHSs have been described previously [18]. Nucleosome fuzziness downstream of TSSs was defined as the coefficient of variance (CV) of the distance between + 1, + 2, + 3 and + 4 nucleosomes, while the position of nucleosomes was defined as the local maximum positions. For the measurement of nucleosomal DNA length, samples were ranked by the deviation of the measurement values to 146 bp in ascending order. For the measurement of nucleosome fuzziness downstream TSSs, samples were ranked based on the measurement values in ascending order. For each of the other four measurements, samples were ranked based on the measurement values in descending order. For each sample, a ‘Pass’ or ‘Fail’ label was assigned for each QC measurement, except for sequencing coverage. The criteria of ‘Pass’ and ‘Fail’ for AA/TT/AT dinucleotide frequency, nucleosomal DNA length, nucleosome depletion at TSSs and enrichment of well-positioned nucleosome arrays at DHSs were defined previously [18]. For nucleosome fuzziness downstream of TSSs, samples with values lower than 0.4 were defined as ‘Pass’, while others were defined as ‘Fail’.

For each cell or tissue type, the high-quality sample was selected via three indicators. First, the total number of ‘Pass’ QC measurements for each sample was defined as the first rank indicator (N_good_ in Fig. 1a). Then, we count the number of QC measurements for each sample such that the rank quantile better than the average rank quantile for the samples comes from the same cell type as the second indicator (C_better_ in Fig. 1a). Third, we calculated the sum of rank quantiles of all QC measurements for each sample as the third indicator (R_better_ in Fig. 1a). The final ranking order for the samples was determined by ordering N_good_, C_better_ and R_better_ successively. For each cell or tissue type, the sample with the top rank was selected as the ‘High Quality Sample’ (Additional file 1: S1 in Fig. 1a).

For each cell or tissue type, the ‘Pooled Sample’ was pooled by samples with high correlation that come from the same cell type. First, we calculated nucleosome occupancy in 1 kb windows throughout the whole genome for each sample. Then, the Pearson correlation coefficients between samples from the same cell type and treatment condition was calculated. Samples with Pearson correlation coefficients higher than 0.6 were pooled as the ‘Pooled Sample’ (Additional file 1: S2 in Fig. 1a).

‘High Quality Sample’ was defined as the referenced nucleosome organization landscape for each cell type and treatment condition, unless the ‘High Quality Sample’ was included in the samples which were used to build the ‘Pooled Sample’. Samples with coverage lower than five-fold were filtered out.

### Nucleosome organization features on TSS and DHS

Here, we defined several nucleosome organization features on TSSs and DHSs and calculated these features on individual gene or DHS for each referenced nucleosome organization map.

The nucleosome array score downstream of the TSS was calculated as the average nucleosome array profile signal from the TSS to the downstream 1 kb region versus the average signal of the whole genome for the given sample. The nucleosome depletion level at a TSS was calculated as the degree of nucleosome occupancy deficiency at the TSS. The maximum nucleosome occupancy at the central 200 bp bin of the site was defined as *N*_*cente*r_, and the maximum nucleosome occupancy surrounding the central bin spanning 200 bp on both sides was defined as *N*_*background*_. If *N*_*center*_ was larger than *N*_*background*_, the nucleosome depletion level of the site was assigned to 0. Otherwise, the nucleosome depletion level of the site was calculated as 1 – *N*_*center*_/*N*_*background*_. The nucleosome occupancy at the TSS was calculated as the average nucleosome occupancy signal at the central 100 bp bin of the TSS.

The + 1 nucleosome, the number of nucleosomes, the linker length and the standard deviation of linker length were calculated as follows. First, the nucleosome occupancy profile for each TSS was scanned and smoothed from the TSS to the downstream 1 kb bin. The local maximal signal was detected, and the relative distance to TSS was defined as candidate of nucleosome positions. If the adjacent nucleosome position was closer than 100 bp, the nucleosome position with higher nucleosome occupancy was maintained. Finally, the nucleosome position downstream of the TSS was defined. The first nucleosome position was defined as the + 1 nucleosome. The linker length was calculated as the average distance between adjacent nucleosome positions subtracting 147 bp, which is the standardized nucleosomal DNA length. The standard deviation of linker length and nucleosome count can be calculated after the nucleosome positions are defined. If the + 1 nucleosome was undetectable, the + 1 nucleosome, linker length and standard deviation of linker length were stored as -1, so that they can be easily recognized. If the number of nucleosomes was less than 2, the linker length and standard deviation of linker length were stored as -1. If the number of nucleosomes was less than 3, the standard deviation of linker length was stored as -1.

The nucleosome organization features on DHSs were calculated in the same way as TSSs. There are two major differences. The nucleosome array score upstream of the DHS was also calculated. The -1 nucleosome, the number of nucleosomes, the linker length and the standard deviation of the linker length 1 kb upstream were calculated by scanning the nucleosome occupancy profile 1 kb upstream of the DHS.

### Prediction of TF binding sites

We collected TF ChIP-seq data from the Cistrome DB database [26] as the actual TF binding profile. Qualified ChIP-seq samples were selected by the QC measurements provided in the database. We scanned the entire genome for significant motif hits for each TF by using BINOCh [27]. For each TF ChIP-seq sample, motif hits overlapping with TF binding peaks were defined as the positive samples (bound sites). The negative samples were defined as other motif hits that did not overlap with any detected peaks in the ChIP-seq sample (unbound sites). All positive and negative samples were used for evaluating the TF binding prediction. A ten-fold cross validation strategy was applied during the model training and testing. The motif score calculated by BINOCh was the first predictor. Nucleosome organization features acted as the second predictor to improve the prediction performance. Here, we include the nucleosome depletion level and nucleosome occupancy on motif hits and the nucleosome array score flanking the motif hits in the prediction model. First, the nucleosome depletion level of the motif hits was calculated in the same way as the nucleosome depletion level on the TSS, while the given site was replaced by the center of the motif hits. Then, nucleosome occupancy on motif hits was calculated as the average nucleosome occupancy at the central 100 bp bin of the sites. The nucleosome array score on the upstream or downstream 1 kb bin of the site was calculated as the average nucleosome array profile signal upstream or downstream of the 1 kb bin of the given site.

A logistic linear regression conducted by a ‘glm’ function in R was performed to match the actual TF binding status (bound or unbound) by scoring motif score and nucleosome organization information. The R package ‘pROC’ was used to evaluate the prediction power by calculating the true-positive rate and true-negative rate with different thresholds, and area under the curve (AUC) scores calculated by the ‘auc’ function in R were used as an indicator to evaluate the performance of the TF binding prediction. The prediction improvement was calculated as AUC (motif score + nucleosome organization information) – AUC (motif score).

## Supplementary Information

**Additional file 1**: Supplementary tables and figures. Table S1 is the summary of MNase-seq data of various cell and tissue types in human for sample filtration. Table S2 is the summary of MNase-seq data of various cell and tissue types in mouse for sample filtration. Table S3 is the summary of ChIP-seq data of TFs used in the TF binding prediction model. Figure S1 shows the summary of QC measurements of all MNase-seq samples collected. Figure S2 shows that nucleosome organization features improve TF binding prediction.

## Data Availability

NUCOME database is available at http://compbio-zhanglab.org/NUCOME. The standardized analysis pipeline CAM is available at https://github.com/TongjiZhanglab/CAM.

## Abbreviations

MNase: Micrococcal nuclease
QC: Quality control
TFs: Transcription factors
NFR: Nucleosome-free regions
TSSs: Transcription start sites
DHSs: DNase hypersensitive sites
ROC: Receiver operating characteristic
AUC: Area under the curve
