# Supplementary material for: NUCOME: A comprehensive database of nucleosome organization referenced landscapes in mammalian genomes
Source: BMC Bioinformatics. 2021 Jun 13;22:321. doi: 10.1186/s12859-021-04239-9 (PMC8201709; doi:10.1186/s12859-021-04239-9)
Supplement: Supplementary file 1 — Additional file 1: Supplementary tables and figures. Table S1 is the summary of MNase-seq data of various cell and tissue types in human for sample filtration. Table S2 is the summary of MNase-seq data of various cell and tissue types in mouse for sample filtration. Table S3 is the summary of ChIP-seq data of TFs used in the TF binding prediction model. Figure S1 shows the summary of QC measurements of all MNase-seq samples collected. Figure S2 shows that nucleosome organization features improve TF binding prediction. [file 12859_2021_4239_MOESM1_ESM.pdf]

Supplementary material

Supplementary Table S1. Summary of MNase-seq data of various cell and tissue types in human for sample filtration

Cell or tissue type	Data count	Cell or tissue type	Data count
BE2C	2	HUVEC	3
brain	1	IMR90	2
Burkitt's lymphoma Raji B cell	5	iPSC	2
CD34	1	K562	5
CD36	2	Lymphoblastoid cell line	14
CD4+ T-cell	6	MCF-7	4
CD8+ T-cell	3	MDA-MB-231	10
Colo829	1	muscle	1
Cultured Kidney Cell	2	NCCIT	1
Fibroblasts	4	Neuroblastoma	2
Granulocyte	8	Sperm	6
H1	6	T-47D	1
H9	2	WA09	1
HEK293 cells	4	WA09-INM	1
HeLa	8	WA09-SMC	1
HN13(TVA)	1	iciHHV6 patient smooth muscle cells	4
hNEC	1		

Supplementary Table S2. Summary of MNase-seq data of various cell and tissue types in mouse for sample filtration

Cell or tissue type	Data count	Cell or tissue type	Data count
2nd generation iPS cells	6	Liver	28
46C	5	Liver, 3-6months	47
AB2.2	2	Liver,21month	2
activated B cell	6	Liver,3month	2
TTF	2	LMPP	2
adult liver	12	Lung Epithelium	2
ALP	2	MEF	12
Astrocytes	1	muscle	1
blastocyst, E3.5	3	NPC	18
BLP	2	Nucleus accumbens cells	6
BMDM	12	Olfactory epithelium	1
brain	1	Thymocytes	16
C2C12	9	Olfactory sensory neuron	1
C2C12 myotubes	1	oligodendrocyte progenitor cells	1
CMTi-1	2	OS25	1
Condensing spermatids	2	Pachytene spermatocytes	5
DRG neurons	2	pre-B cell line B3	2
E14	18	pre-iPSC	2
E14Tg2a	6	pro-B	2
Erythroblasts	2	quadriceps muscle	3
ESC	22	R1	5
Fetal Liver	5	resting B cell	6
Germline stem cells	2	Round spermatids	2
haftl derived C10 cells	4	round-elongating spermatids	4
heart	1	Small pre-B	2
Hepa-1c1c7	4	Sperm	9
HSC	2	Spermatocytes	2
Hypothalamus	2	Testis	2
iPSC	2	Tg2a-mitosis-A	9
iPSC.TTF	2	Tg2a-mitosis-M	9
isolated adult ventricular cardiomyocytes	4	Olfactory epithelium neural progenitor cell line OP27	8
isolated ventricular cardiomyocytes	9	adenocarcinoma cell line 3134	4
J1	13	V6.5	3
kidney	1	VSMC	4
Large pre-B	2		

Supplementary Table S3. Summary of ChIP-seq data of TFs used in the TF binding prediction model

Cell line	TF	GEO accession ID	Cell line	TF	GEO accession ID
GM12878	CTCF	GSM489290	K562	CEBPB	GSM935499
GM12878	EGR1	GSM803434	K562	CTCF	GSM624080
GM12878	ELF1	GSM803496	K562	E2F6	GSM935597
GM12878	MAX	GSM935518	K562	EGR1	GSM803414
GM12878	NFYB	GSM935507	K562	ELF1	GSM803494
GM12878	PAX5	GSM803362	K562	ETS1	GSM803442
GM12878	RUNX3	GSM1010893	K562	FOS	GSM610336
GM12878	YY1	GSM803406	K562	GABPA	GSM803524
H1	CTCF	GSM624077	K562	MAFF	GSM935520
H1	E2F6	GSM1010899	K562	MAX	GSM935344
H1	GABPA	GSM803424	K562	MAZ	GSM935337
H1	SP4	GSM1010743	K562	USF1	GSM803441
H1	USF1	GSM803426	K562	YY1	GSM803470
H1	YY1	GSM803513	MCF-7	CEBPB	GSM1010889
HeLa	CTCF	GSM624078	MCF-7	CTCF	GSM614615
IMR90	CEBPB	GSM935519	MCF-7	E2F1	GSM699989
IMR90	CTCF	GSM935404	MCF-7	ELF1	GSM1010764
IMR90	MAZ	GSM1003613	MCF-7	MAX	GSM1010863

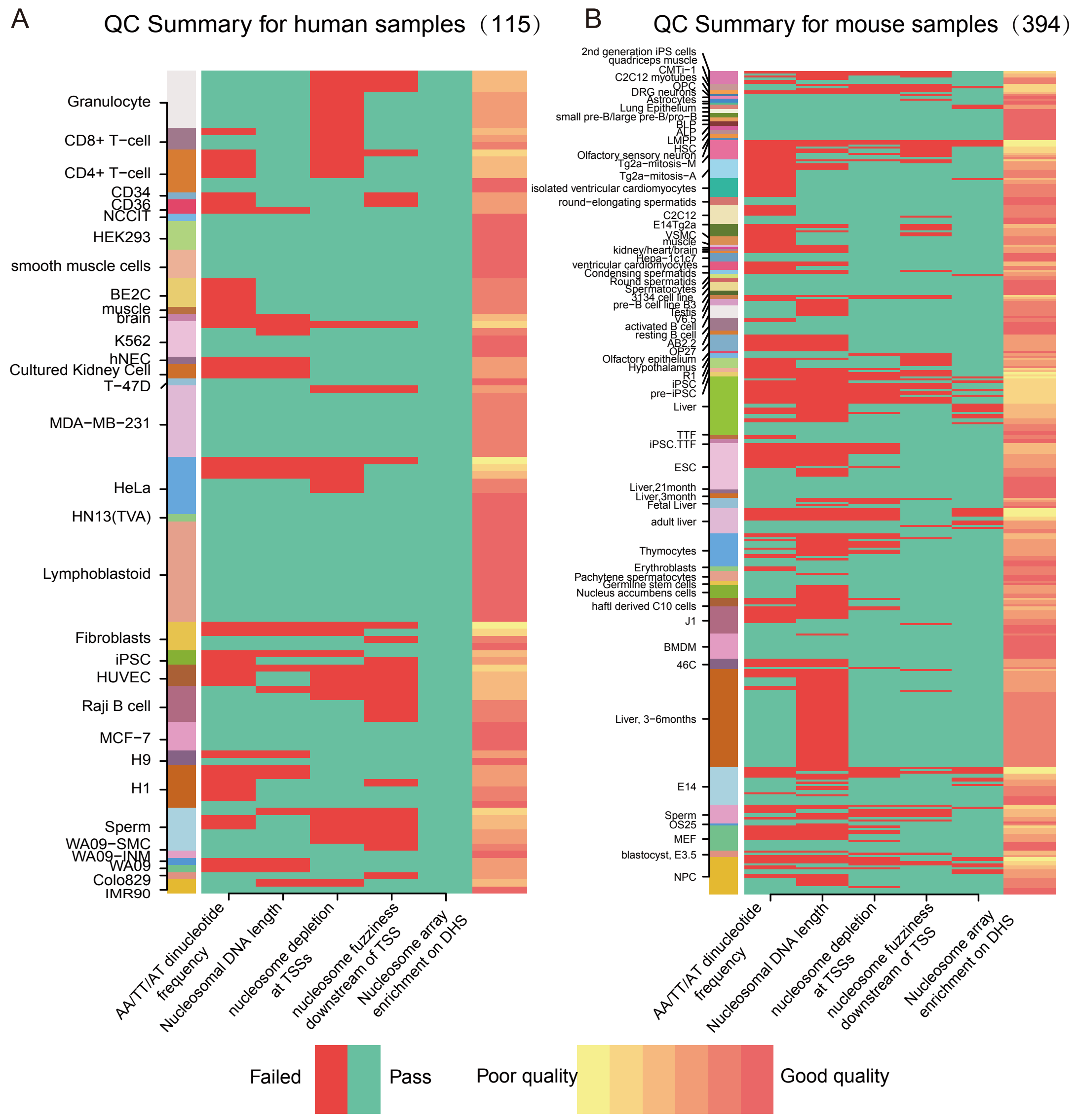

Figure S1. Summary of QC measurements of all MNase-seq samples collected.

A. QC assessments of five QC measurements except for sequencing coverage of all MNase-seq data in human. Samples in different cell or tissue types are labeled with different colors at the left grids. The data quality of each sample is labeled 'Pass' or 'Fail' for each QC measurement. Green grids represent 'Pass' in QC, while red grids represent 'Fail'. The total number of 'Pass' QC measurements of each sample are displayed at the right grids. Red grids exhibit better data quality considering the six QC measurements, while yellow grids indicate worse data quality. **B**. QC assessments of five QC measurements except for sequencing coverage of all MNase-seq data in mouse.

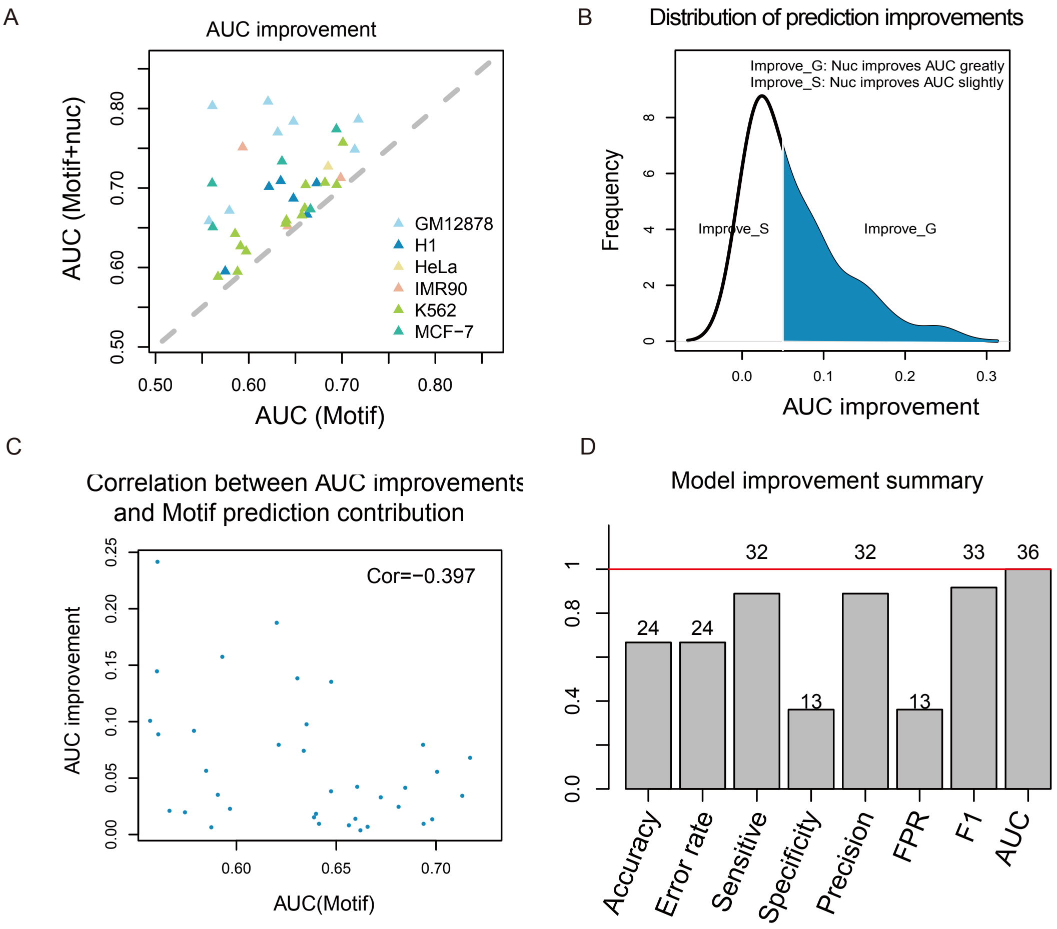

Figure S2. Nucleosome organization features improve TF binding prediction.

A. Scatter plot of the improvement of prediction power by introducing nucleosome organization features in the prediction model. The x-axis represents the AUC score by using the DNA motif score only. The y-axis represents the AUC score by using both DNA motif score and nucleosome organization information. Different colors represent the TFs in various cell and tissue types. The gray dotted line indicates that the AUC scores of the two predictions are equal. **B**. Distribution of AUC score improvements by introducing nucleosome organization features in the prediction model. The 'Improve_G' group includes TFs exhibiting at least a 0.05 improvement, while the 'Improve_S' group includes TFs with less than 0.05 AUC score improvement. **C**. Scatterplot revealing the significantly negative correlation between prediction improvement by introducing nucleosome organization features and prediction performance by DNA motif score only. The x-axis represents the AUC

score by using the DNA motif score only. The y-axis represents the AUC score improvements by introducing nucleosome organization features. **D**. Summary of prediction improvement of models by counting the number of models with improved assessment indicators, including accuracy, error rate, sensitve, specificity, precision, FPR, F1 score and AUC of ROC space.
